# Assessing the win–win situation of forage production and soil organic carbon through a short-term active restoration strategy in alpine grasslands

**DOI:** 10.3389/fpls.2023.1290808

**Published:** 2024-01-11

**Authors:** Yan Wang, Zhicheng Wang, Yukun Kang, Zhiming Zhang, Duanhong Bao, Xiaomei Sun, Junhu Su

**Affiliations:** ^1^ College of Grassland Science, Gansu Agricultural University, Lanzhou, China; ^2^ Key Laboratory of Grassland Ecosystem, Ministry of Education, Gansu Agricultural University, Lanzhou, China; ^3^ Gansu Agricultural University-Massey University Research Centre for Grassland Biodiversity, Gansu Agricultural University, Lanzhou, China; ^4^ College of Resource and Environmental Science, Gansu Agricultural University, Lanzhou, China

**Keywords:** grassland degradation, ecological restoration, short-term, soil properties, plant

## Abstract

**Introduction:**

Grassland degradation has seriously affected the ecological environment and human livelihood. To abate these, implementing effective management strategies to restore and improve the service functions and productivity of degraded grasslands is crucial.

**Methods:**

To evaluate the influences of restoration measures combined with different grazing intensities on short-term (1 year) grassland restoration, the changes in soil physicochemical properties, as well as plant traits under restoration measures of different grazing intensities, reseeding, and fertilization, were analyzed.

**Results:**

Soil organic carbon (SOC) increased to varying degrees, whereas available nutrients decreased under all combined restoration measures. Reseeding, alone and in combination with fertilization, substantially increased SOC, improved grassland vegetation status, and enhanced grassland productivity. The aboveground biomass of Gramineae and the total aboveground biomass increased under the combined restoration measures of transferring livestock out of the pasture 45 days in advance, reseeding, and fertilization (T4). Redundancy analysis revealed a strong correlation between grassland vegetation characteristics, SOC, and available potassium. Considering soil and vegetation factors, the short-term results suggested that the combination measures in T4had the most marked positive impact on grassland restoration.

**Discussion:**

These findings offer valuable theoretical insights for the ecological restoration of degraded grasslands in alpine regions.

## 1 Introduction

Grasslands are important components of terrestrial ecosystems that maintain human livelihoods and national ecological security (Gomez-Casanovas et al., 2021). In the last few years, the overuse of grassland resources due to increases in population and animal husbandry and a chronic lack of scientific management of grassland resources has severely deteriorated the ecological environment and productivity of the grassland (Zhang et al., 2019; Bardgett et al., 2021). Previous research claims that nearly 39.06% of grasslands globally have some form of degradation (Liu et al., 2019). Although the overall trend in grassland degradation has been curbed in the last 10 years, grassland degradation in some areas has become more serious, and the productive capacity of some grasslands has been lost completely (Hou et al., 2022). The world’s largest alpine grassland ecosystem is found on the Qinghai–Tibet Plateau, which is significant to the development of local animal husbandry, water conservation, and environmental security (Liu et al., 2021). Since the 1970s, the development of animal husbandry on the Qinghai–Tibet Plateau has resulted in overgrazing and a sharp reduction in grassland (Zhao et al., 2022). Natural grassland vegetation coverage and productivity have decreased, inedible forage, poisonous plants, and weeds have increased, and some seriously degraded grasslands have even transformed into “bare lands”. This seriously threatens local ecological security and the development of animal husbandry (Shang et al., 2008; Dong, 2023). Therefore, for the sustainable development of the economy, society, and the ecological environment of local pastoral areas, the restoration and management of degraded grasslands have become critical.

Plant–soil interactions determine the stability of grassland formation and development. Two basic characteristics of community stability, species diversity and productivity, are the core indices of the grassland ecosystem (Pennekamp et al., 2018). Species diversity reflects the complex relationship between organisms and their environment as well as the richness of biological resources. The aboveground biomass mirrors the vegetation characteristics and productivity of grasslands and maintains ecosystem diversity (Hector et al., 1999; Dietrich et al., 2023). Grassland degradation contributes to the reverse transformation of the structure and function of grassland ecosystems, simultaneously weakening grassland productivity, which leads to biodiversity loss and community destabilization (Dong et al., 2018; Berdugo et al., 2020). Grassland degradation includes vegetation and soil degradation. Vegetation degradation manifests as decreases in biomass, coverage, the proportion of edible herbage, species diversity, and stability and an increase in poisonous plants and weeds. Soil degradation is manifested in variations in soil physicochemical properties and structure and the deterioration of microbial and enzymatic activities (Xie et al., 2018; Peng et al., 2019; Su et al., 2023). Vegetation degradation is directly caused by soil degradation. Vegetation growth and development are heavily dependent on the supply of soil nutrients. Therefore, vegetation growth is restricted by soil fertility to a certain extent, indicating mutual influence between these factors (Wu et al., 2020; Kooch et al., 2022).

Restoration measures for degraded grasslands mainly include enclosures, restoration of farmland to grassland, turf cutting, no-tillage reseeding, and fertilization. In view of the existing difficulties in the restoration of degraded grasslands in different areas, restoration methods also differ (Gu et al., 2022; Wu et al., 2023) —for example, some researchers have taken measures, such as enclosure, trench excavation, and fish-scale pit construction, to restore degraded grasslands to their typical state in the hill area of the Loess Plateau (Han et al., 2015). Different measures for restoring degraded grasslands have also been studied. In mildly degraded grasslands, fencing, rodent control, and varying grazing intensities and durations have been adopted (Choi, 2007). For moderately degraded grasslands, measures such as reseeding and fertilization have been adopted (Wu et al., 2023). Artificial grassland restoration measures have been adopted in severely and extremely degraded grasslands (Liu et al., 2017; Liu et al., 2022). Several investigations have found that different combinations of measures have different restoration effects on degraded grasslands (Yin et al., 2021; Duan et al., 2022). Compared to single grassland restoration measures, combined measures have a more apparent improvement on degraded grasslands (Wei et al., 2022; Wu et al., 2023).

Previous studies on the restoration of degraded grassland were conducted in grazing-prohibited sites, which focus on the long-term grassland restoration but not on short-term productivity, animal husbandry development, and other livelihood issues (Bai et al., 2020; Dietrich et al., 2023). We used the grassland of Gannan Prefecture to explore the variations in soil properties and vegetation characteristics under different combined restoration measures with different grazing intensities and the relationships between them. The objective was to clarify the short-term effects of different combined measures of restoration on degraded grasslands and determine which restoration measure with regional characteristics is suitable for local traditional production modes. The results of this study may provide new ideas and theoretical references for the future comprehensive solution to production, ecological, and people’s livelihood problems.

## 2 Methods

### 2.1 Study sites

**Figure 1:**
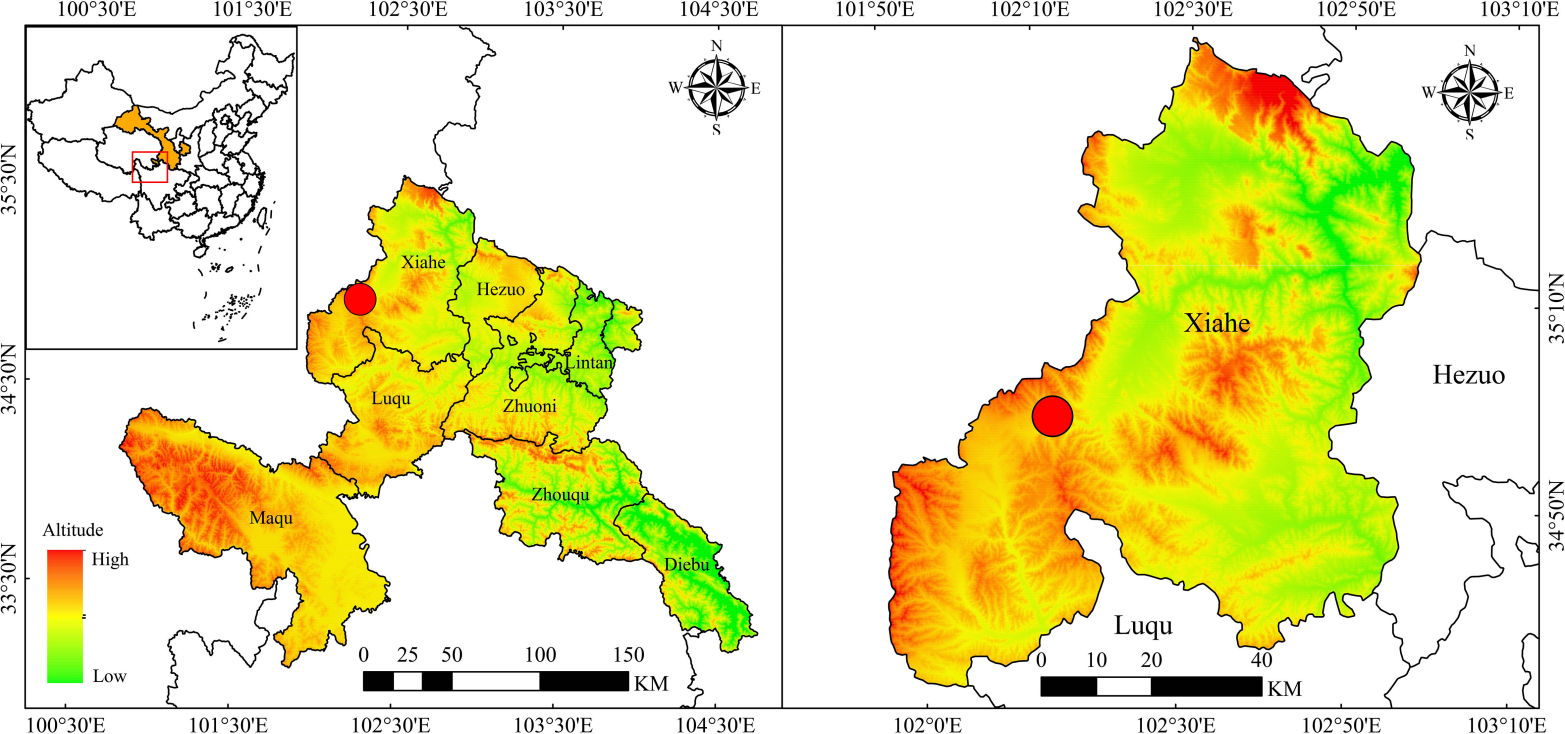
Location of the study site in Xiahe County, Gannan Prefecture. The red circle represents the study site.

Our experiment was conducted at Sangke grassland (34°51′N–34°52′N, 102°04′E–102°07′E) in Xiahe County, Gannan Prefecture (**Figure 1**). The altitude is 3,200–3,500 m, the mean annual air temperature is 2.6°C, and the mean annual precipitation is 500 mm. Rainfall mainly occurs from July to September, when grass growth is strong. The climate is cold and humid, the temperature varies widely from day to night, rain and heat occur during the same season, and the vertical temperature differential is substantial. Absolute non-frost periods are not observed. The vegetation growing season is 120–140 days. The soil type is subalpine meadow soil with alpine and mountain meadow grasslands.

The excellent herbage in grassland vegetation predominantly included *Elymus nutans*, *Carex lanceolata*, *Poa pratensis*, *Lolium perenne*, *Festuca ovina*, *Polygonum viviparum*, *Kobresia humilis*, and *Trigonella ruthenica*. The poisonous plants and weeds included *Potentilla bifurca*, *Oxytropis kansuensis*, *Leontopodium leontopodioides*, *Gentiana macrophylla*, *Thalictrum aquilegiifolium*, *Achnatherum inebrians*, *Gentiana dahurica*, *Delphinium grandiflorum*, *Ligularia virganrea*, and *Pedicularis kansuensis*.

### 2.2 Experimental design

**Figure 2:**
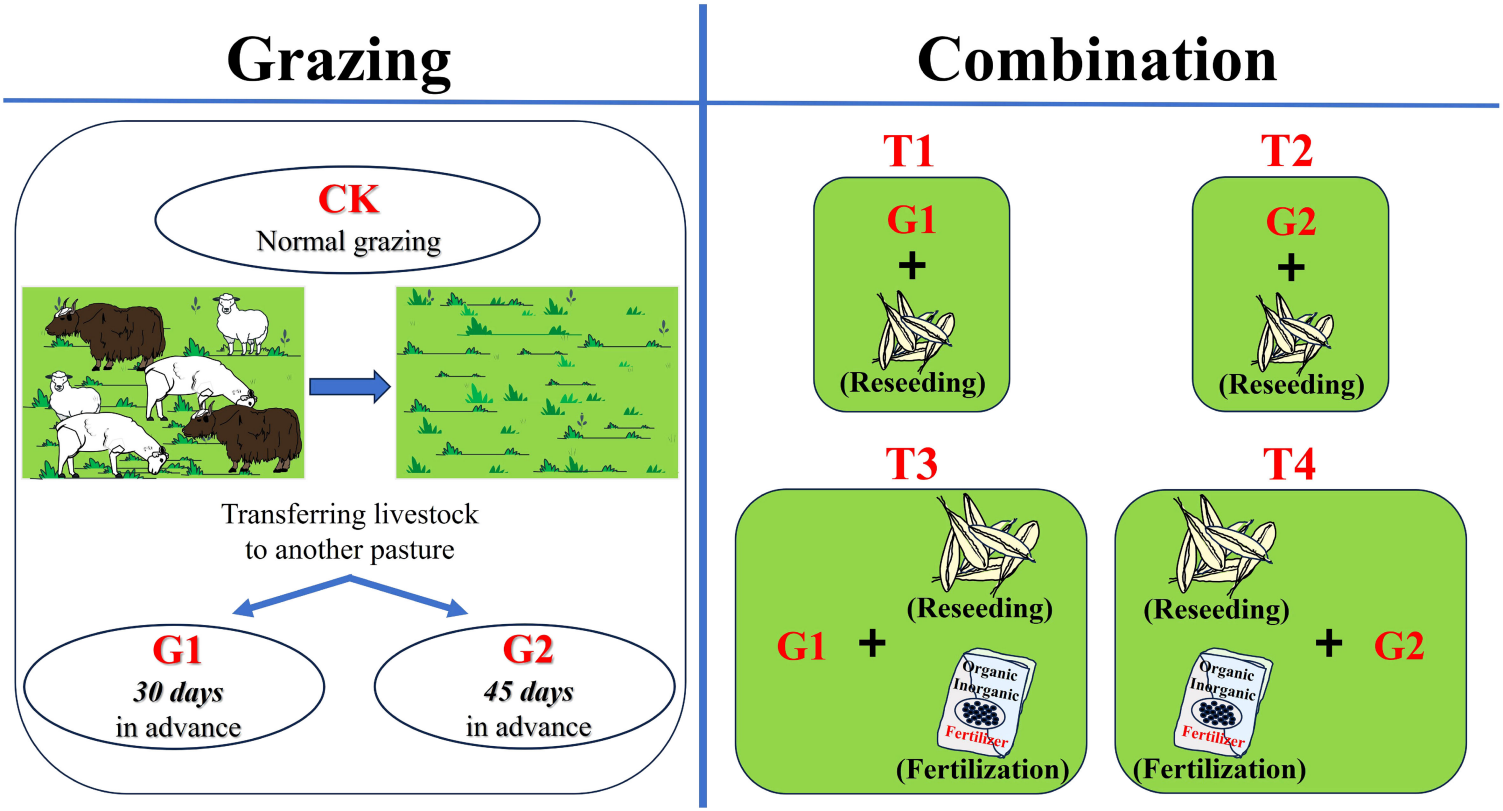
Diagram of different restoration measures in degraded alpine grasslands.

Three grazing intensities and four combined restoration measures were used at the study sites (**Figure 2**). Our study was conducted under the conditions of a certain area of the grassland and the number of livestock (2.5 standard sheep unit/hm^2^). The specific times of the three grazing treatments were as follows: CK: normal grazing on May 23, 2020; G1: transfer of livestock out of the pasture 30 days in advance on April 23, 2020; and G2: transfer of livestock out of the pasture 45 days in advance on April 7, 2020. The four restoration measures were as follows: T1: combination of G1 and reseeding; T2: combination of G2 and reseeding; T3: combination of G1, reseeding, and fertilization; and T4: combination of G2, reseeding, and fertilization.

The native grass species used for the mechanical no-tillage reseeding were *E. nutans*, *Poa crymophila*, and *L. perenne.* The seeding rates of the three grasses were 30 kg/hm^2^ *E. nutans*, 15 kg/hm^2^ *P. crymophila*, and 15 kg/hm^2^ *L. perenne*. The three types of grass were mixed during sowing.

An organic–inorganic compound fertilizer was selected. The fertilizer nutrients were as follows: (B + Za + Fe + Mo) 0.1%, (S + Ca + Mg) 3%, (N + P_5_O_2_ + K_2_O) 17%, pH = 6.0–6.5, and organic matter content 30%. The application rate of organic and inorganic compound fertilizers was 1,200 kg/hm^2^. Mechanical fertilization, disc harrow cutting, covering of the soil, and other processes were required before sowing to ensure that the fertilizer was fully embedded within the soil.

### 2.3 Vegetation and soil sampling

Field survey sampling was conducted in August 2020. The experiment included four sites with different combined restoration measures and three sites with different grazing intensities. Every site was 5 hm^2^. We selected three similar plots (0.5 m × 0.5 m) at each site randomly. The coverage and natural height of each species were measured. Within each plot, all plants, including both live and standing dead biomass, were clipped to ground level inside each plot to calculate the total aboveground biomass (AGB). Within each site, soil samples were collected from each of the three replicated plots (*n* = 3) at three different depths (0–10, 10–20, and 20–30 cm), and a total of five soil cores with a diameter of 5 cm were randomly sampled at each depth. We formed a composite sample, which was uniformly mixed using the soil samples from the same depth at each site.

We used the over-drying method to detect the soil water content (SWC) (Thomasson, 1978). The litter, plant roots, and stones in the soil samples were removed manually. Then, the soil samples were air-dried. The soil was ground fully and passed through a sieve with a pore size of 0.25 mm. The soil pH was measured using a pH meter (Sartorlus, Beijing, China). The dichromate oxidation method was used to measure the soil organic carbon (SOC). The soil total phosphorus (TP) was measured using the HClO_4_–H_2_SO_4_ method. The Kjeldahl method was used to determine the soil total nitrogen (TN) (Bao, 2000). The available potassium (AK) and total potassium (TK) were detected using a flame photometer after digestion with NaOH and extraction with CH_3_COONH_4_, respectively (Han et al., 2022). The available soil nitrogen (AN) content was identified using an alkali–hydrolysis reduction diffusion method (Bao, 2000). The ammonium bicarbonate method was used to extract the available soil phosphorus (AP) (Zhang et al., 2018).

### 2.4 Statistical analyses

The experimental data were processed using SPSS v. 26.0 (IBM SPSS Inc., Chicago, IL, USA), and the mean and standard deviation were used to express the measured outcomes. One-way analysis of variance (ANOVA) was performed to identify the physicochemical properties of the soil as well as the vegetation characteristics (height, coverage, and aboveground biomass of vegetation) under different ecological restoration measures. OriginPro v. 2021 (OriginLab Corporation, Northampton, MA, USA) was used for drawing. The relationship between grassland vegetation characteristics and soil factors was determined using redundancy analysis (RDA) using the “vegan” R package. The Monte Carlo displacement test (999 displacement cycles) was used to explore the significance of soil factors on vegetation change, and the Envfit function (999 displacement cycles) was used to confirm the relationship between various soil factors and vegetation change (Oksanen et al., 2019). Asterisks indicate the significance level between soil factors and plants (**P < 0.01).

## 3 Results

### 3.1 Effects of soil water content and pH under different restoration measures

**Figure 3:**
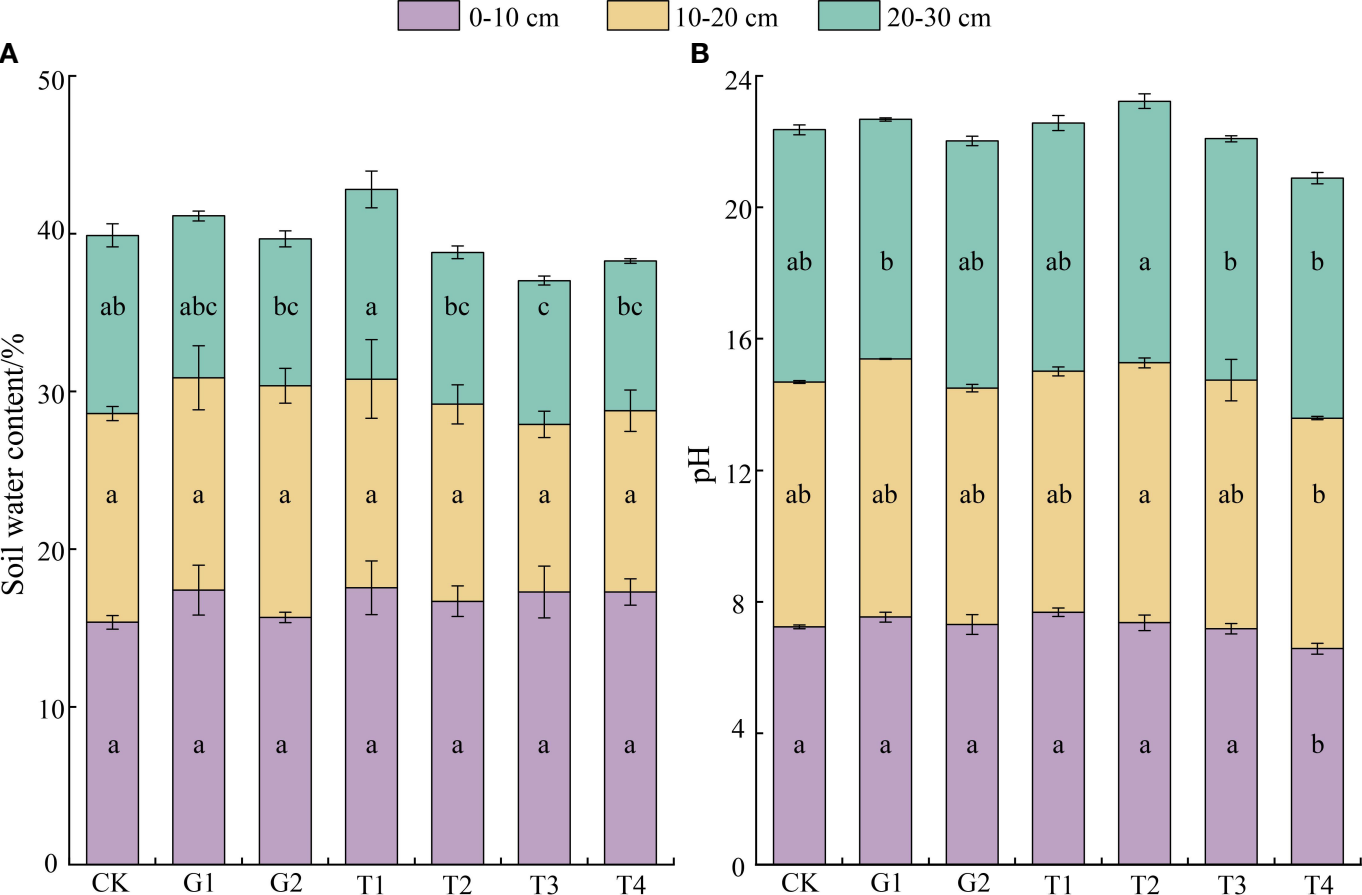
Soil water content and pH under different restoration measures (*n* = 3 in each site). **(A)** Soil water content; **(B)** Soil pH. Different lowercase letters in the same color indicate significant differences between treatments at the same soil depth (ANOVA, significance level = 0.05). CK, normal grazing; G1, transfer of livestock out of pasture 30 days in advance; G2, transfer of livestock out of pasture 45 days in advance; T1, combination of G1 and reseeding; T2, combination of G2 and reseeding; T3, combination of G1, reseeding, and fertilization; T4, combination of G2, reseeding, and fertilization.

The SWC at three soil layer depths under different restoration measures, from highest to lowest, was 0–10 > 10–20 > 20–30 cm. In all three soil layers, the SWC of the T1 site was the highest, whereas that of T3 was the lowest. The SWC showed differences only in the third soil layer, and it was higher in T1 than in the other combination sites (*P* < 0.05) (**Figure 3A**). A marked discrepancy appeared in soil pH between sites T2 and T4. The soils at all the sites were weakly alkaline (**Figure 3B**).

### 3.2 Effects of soil nutrients under different restoration measures

**Figure 4:**
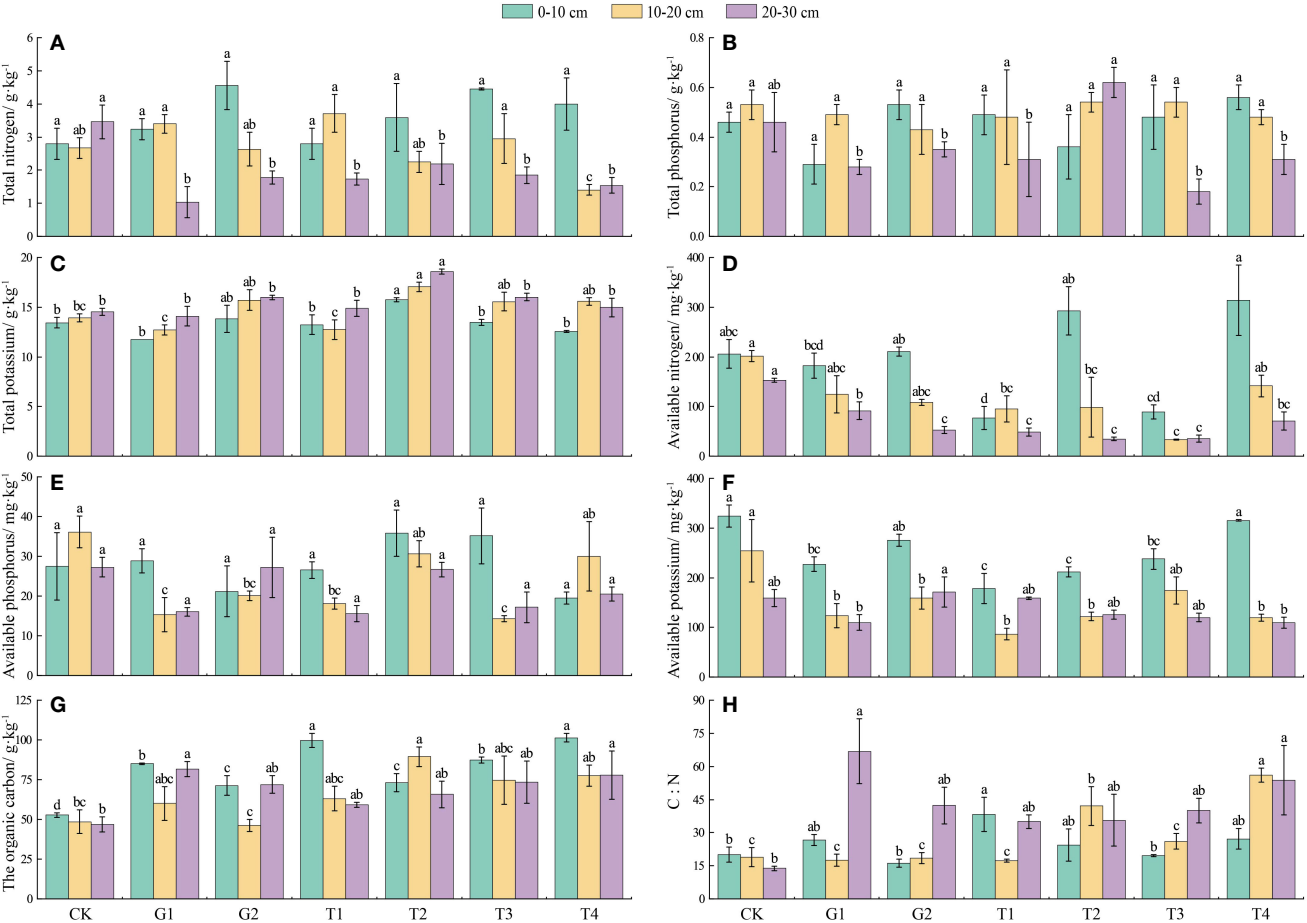
Soil nutrients under different restoration measures (*n* = 3 in each site). **(A)** Soil total nitrogen; **(B)** Soil total phosphorus; **(C)** Soil total potassium; **(D)** Soil available nitrogen; **(E)** Soil available phosphorus; **(F)** Soil available potassium; **(G)** Soil organic carbon; **(H)** C:N. Different lowercase letters in the same color indicate significant differences between treatments at the same soil depth (ANOVA, significance level = 0.05). CK, normal grazing; G1, transfer of livestock out of pasture 30 days in advance; G2, transfer of livestock out of pasture 45 days in advance; T1, combination of G1 and reseeding; T2, combination of G2 and reseeding; T3, combination of G1, reseeding, and fertilization; T4, combination of G2, reseeding, and fertilization.

A comparison of the differences in soil nutrients at different sites revealed that the TP, TN, TK, AK, and AP contents in the soil decreased and then increased as grazing intensified. Under different restoration measures, no differences were observed in soil AN in the first soil layer among the different grazing sites (*P* > 0.05), but the soil AN at the T2 and T4 sites was higher than that at the T1 and T3 sites, respectively (*P* < 0.05) (**Figure 4A**). The soil AP content did not change in the first and third soil layers (*P* > 0.05); however, the soil AP content at the T2 and T4 sites in the second soil layer was higher than that at the T1 and T3 sites, respectively (*P* < 0.05) (**Figure 4B**). Compared with the T3 site, the soil AK in the first soil layer was higher and the TN in the second soil layer was lower at the T4 site (*P* < 0.05). The AK in the second soil layer and TN in the third soil layer at the CK site were the highest among all three grazing sites (*P* < 0.05) (**Figures 4C, D**). There was no difference in soil TP in the first and second soil layers (*P* > 0.05), and the TP in the third soil layer at the T2 site was higher than that at the T1 site (*P* < 0.05) (**Figure 4E**). The soil TK content at the T2 site in the first and third soil layers was higher than that at the T1 site *(P* < 0.05) (**Figure 4F**). The SOC content at the CK site was the lowest of all three grazing sites (**Figure 4G**). The soil C:N ratio in the first soil layer was higher at the T1 site than at the CK and G2 sites (*P* < 0.05). In the second soil layer, the soil C:N at the T2 and T4 sites was higher than that at the T1 and T3 sites, respectively (*P* < 0.05). The C:N at the G1 site was higher than that at the CK site in the third soil layer (*P* < 0.05), and that at the CK site was the lowest among all three grazing sites (**Figure 4H**).

### 3.3 Effects of aboveground vegetation biomass under different restoration measures

**Figure 5:**
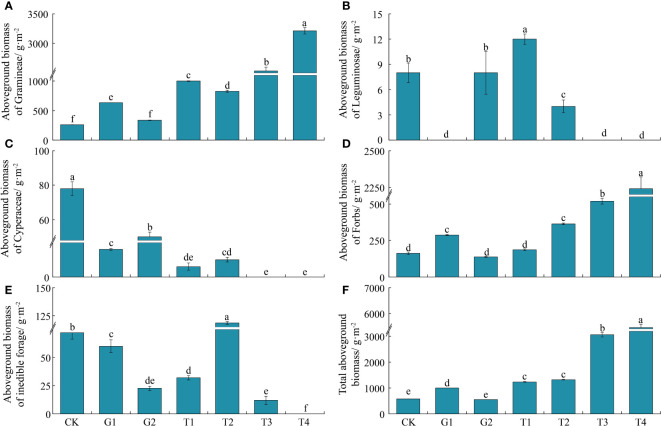
Aboveground vegetation biomass under different restoration measures (*n* = 3 in each site). **(A)** Aboveground biomass of Gramineae; **(B)** Aboveground biomass of Leguminosae; **(C)** Aboveground biomass of Cyperaceae; **(D)** Aboveground biomass of Forbs; **(E)** Aboveground biomass of inedible forage; **(F)** Total aboveground biomass. Different lowercase letters indicate significant differences between treatments (ANOVA, significance level = 0.05). CK, normal grazing; G1, transfer of livestock out of pasture 30 days in advance; G2, transfer of livestock out of pasture 45 days in advance; T1, combination of G1 and reseeding; T2, combination of G2 and reseeding; T3, combination of G1, reseeding, and fertilization; T4, combination of G2, reseeding, and fertilization.

The aboveground vegetation biomass under different restoration measures was compared. No reduction in the G2 site was found compared with the CK site in the aboveground biomass of Gramineae (*P* > 0.05), but that at the other sites varied significantly (*P* < 0.05). The aboveground biomass of Gramineae, from highest to lowest, was T4 > T3 > T1 > T2 > G1 > G2 > CK (**Figure 5A**). There were no Leguminosae at the G1, T3, and T4 sites, and the aboveground biomass of Leguminosae at the T1 site was the highest among the remaining four sites (*P* < 0.05) (**Figure 5B**). Cyperaceae did not appear at sites T3 or T4. The aboveground biomass of cyperaceous plants at the CK and G2 sites was higher than that at the T1 and T2 sites (*P* < 0.05) in the following order: CK > G2 > G1 > T2 > T1 (**Figure 5C**). The aboveground biomass of Forbs at the T3 and T4 sites was higher (*P* < 0.05), and that at the G1 site was the highest of all three grazing sites (*P* < 0.05) (**Figure 5D**). No inedible herbage appeared at the T4 site. The aboveground biomass of inedible herbage was highest at the T2 site (*P* < 0.05), and that at the CK site was the highest (*P* < 0.05) (**Figure 5E**). The AGB at the T3 and T4 sites was higher than that of the other two combined restoration sites (*P* < 0.05). The AGB was the highest at the G1 site among the three different grazing sites (*P* < 0.05) (**Figure 5F**).

**Figure 6:**
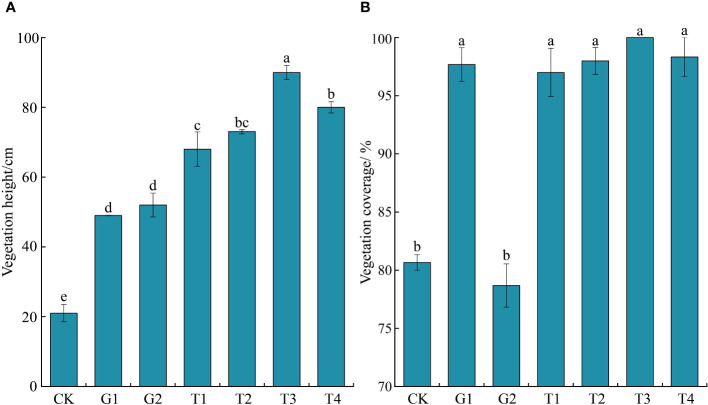
Vegetation height and coverage under different restoration measures (*n* = 3 in each site). **(A)** Vegetation height; **(B)** Vegetation coverage. Different lowercase letters indicate significant differences between treatments (ANOVA, significance level = 0.05). CK, normal grazing; G1, transfer of livestock out of pasture 30 days in advance; G2, transfer of livestock out of pasture 45 days in advance; T1, combination of G1 and reseeding; T2, combination of G2 and reseeding; T3, combination of G1, reseeding, and fertilization; T4, combination of G2, reseeding, and fertilization.

The vegetation height at the CK site was the lowest, and this increased to varying degrees at the other treatment sites. Compared with all three sites with different grazing intensities, both reseeding and the combination of reseeding and fertilization increased the vegetation height in the degraded grasslands (*P* < 0.05) (**Figure 6A**). The vegetation coverage at the CK and G2 sites was lower than that at the G1 and four combined restoration sites (*P* < 0.05) (**Figure 6B**).

### 3.4 RDA of vegetation characteristics and soil factors

**Figure 7:**
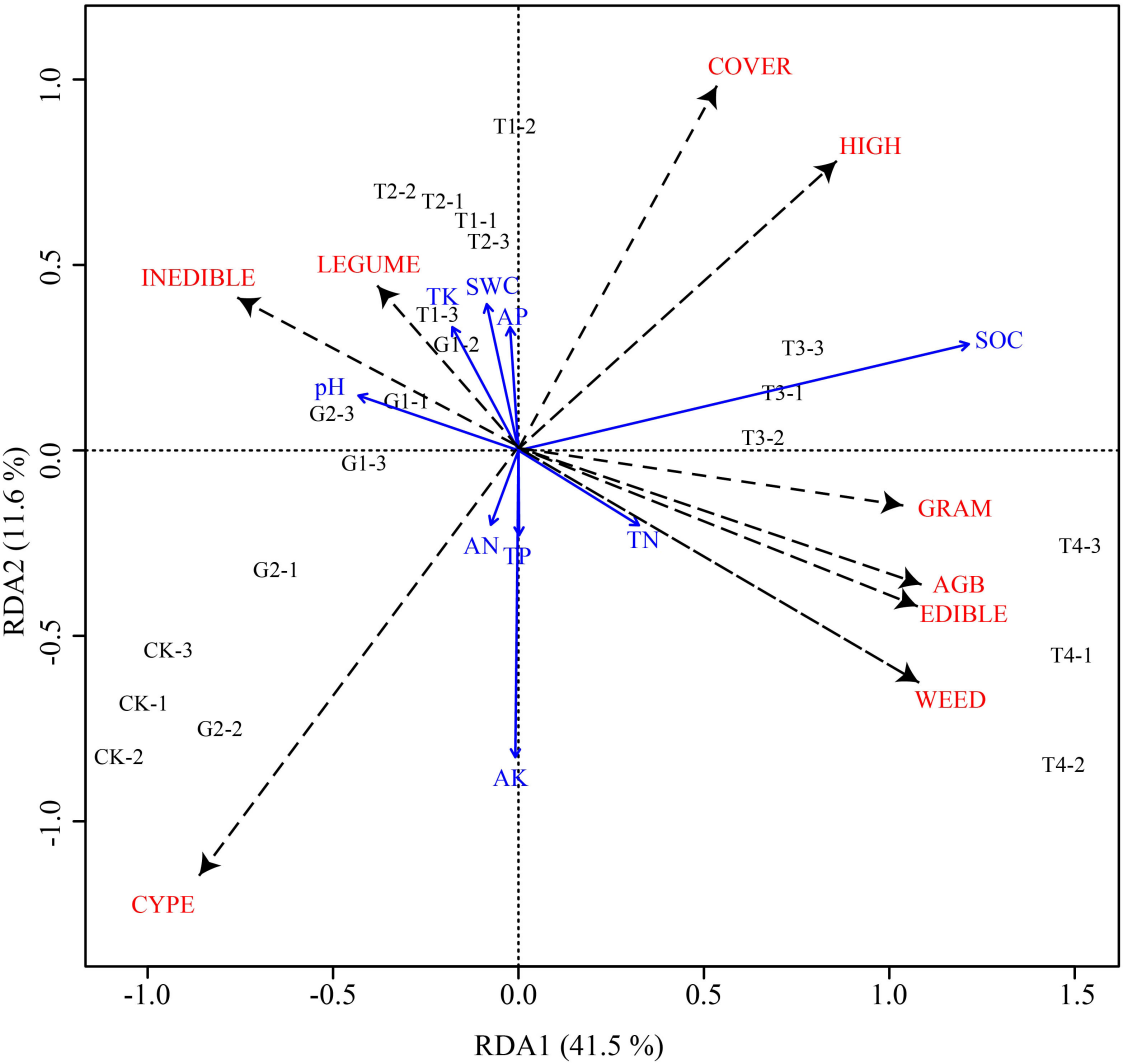
Redundancy analysis (RDA) ranking of vegetation characteristics and soil factors. SWC, soil water content; pH, soil pH; SOC, soil organic carbon; TN, total nitrogen; TP, total phosphorus; TK, total potassium; AN, available nitrogen; AP, available phosphorus; AK, available potassium; LEGUME, aboveground biomass of Leguminosae; HIGH, vegetation height; INEDIBLE, aboveground biomass of inedible forage; CYPE, aboveground biomass of Cyperaceae; COVER, vegetation coverage; WEED, aboveground biomass of Forbs; GRAM, aboveground biomass of Gramineae; AGB, total aboveground biomass; EDIBIO, aboveground biomass of edible forage; CK, normal grazing; G1, transfer of livestock out of pasture 30 days in advance; G2, transfer of livestock out of pasture 45 days in advance; T1, combination of G1 and reseeding; T2, combination of G2 and reseeding; T3, combination of G1, reseeding, and fertilization; T4, combination of G2, reseeding, and fertilization.

The RDA indicated that the first two ranking axes (RDA1 and RDA2) explained 41.50% and 11.60% of the vegetation characteristic changes, respectively, and soil factors explained 53.10% of the vegetation characteristic changes, indicating that RDA1 and RDA2 explained the correlation between soil factors and vegetation features well. A Monte Carlo permutation test was used to identify the variation in soil factors and vegetation characteristics, and the result was significant (*P* < 0.05) (**Figure 7**; **Table 1**).

**Table 1:** Redundancy analysis (RDA) results of vegetation characteristics and soil factors.

Parameter	RDA1	RDA2
Eigenvalues	3.735	1.044
Percentage change in soil factor (%)	41.500	11.600
Cumulative percentage change in soil factor (%)	41.500	53.100
Soil factor–percentage change in vegetation characteristics (%)	66.700	18.650
Soil factor–percentage cumulative change of vegetation characteristics (%)	66.700	85.350
Monte Carlo replacement test	*P* = 0.038	

We used the *envfit* function to explore the relationships between soil properties and changes in vegetation characteristics. In the RDA ranking correlation coefficient, *r* ^2^ represents the determining coefficient of the explanatory variable (soil factors) for vegetation characteristics. The correlations between the soil factors and vegetation change were ranked as follows: SOC > AK > SWC > TK > AP > pH > TN > TP > AN. Organic carbon and available potassium were strongly correlated with changes in grassland vegetation characteristics (*P* < 0.01) (**Table 2**).

**Table 2:** Redundancy analysis (RDA) ranking of correlation coefficients between vegetation characteristics and soil factors.

Soil factor	RDA1	RDA2	*r* ^2^	*Pr* (> *r*)
SWC	-0.141	0.990	0.115	0.342
pH	-0.879	0.476	0.078	0.499
SOC	0.939	0.344	0.552	0.001**
TN	0.715	-0.699	0.065	0.545
TP	0.009	-0.999	0.038	0.727
TK	-0.327	0.945	0.091	0.444
AN	-0.226	-0.974	0.031	0.759
AP	-0.048	0.998	0.079	0.489
AK	-0.002	-1.000	0.494	0.002**

SWC, soil water content; pH, soil pH; SOC, soil organic carbon; TN, total nitrogen; TP, total phosphorus; TK, total potassium; AN, available nitrogen; AP, available phosphorus; AK, available potassium.

**Significance level, *P* < 0.01.

## 4 Discussion

### 4.1 Responses of soil physicochemical properties to different restoration measures

Grassland degradation can directly or indirectly change the soil nutrients and environment of grassland ecosystems, while soil moisture greatly affects grassland productivity (Deng et al., 2016). The first part of this study revealed that SWC was relatively high under moderate grazing conditions (G1) and relatively low under normal grazing conditions (CK), and soil pH did not change substantially among all restoration measures. Overgrazing and grassland degradation can reduce the SWC (Drewry et al., 2008). Trampling by grazing livestock reduces soil porosity; once large pores are lost, water infiltration and SWC decrease (Sanjari et al., 2008). In addition, the decrease in vegetation coverage caused by grassland degradation affects surface evapotranspiration and infiltration, resulting in a decrease in SWC. The relatively high SWC in the 0–20-cm soil layer at all restoration sites was probably due to increased grassland vegetation coverage and decreased surface evaporation caused by moderate grazing and reseeding. The increase in subsurface biomass in the soil would also enhance the water storage capacity of soil (Wang et al., 2023).

pH is an index used to measure soil acidity and alkalinity. A suitable pH is conducive to vegetation growth (Liu et al., 2017). In the current study, restoration measures had a small impact on soil pH in the short term, and soil pH among the different treatments was similar. However, the pH was relatively low in the first soil layer at the T4 site. Fertilization and reseeding reduced the evaporation of soil water, and salt accumulation did not occur in the surface layer, which decreased the pH (Duan et al., 2022). An increase in grazing intensity also leads to an increase in pH (Attard et al., 2008). The T4 site combined light grazing with reseeding and fertilization, which may be a reason for the decrease in soil pH.

Soil nutrients form the basis for plant growth and development. They also play a decisive role in grassland ecosystems (Li et al., 2021). Soil nutrients differed under the different grasslands and restoration measures. With an increase in grazing intensity, the SOC content first increased and then decreased. In contrast, the TN, TP, TK, AP, and AK contents first decreased and then increased. In the case of moderate grazing, the quality of topsoil and litter is improved by the trampling effect of livestock and fecal and urinary regression (Chen et al., 2020). The activities of soil microorganisms and nutrient cycling at the ecological interface between vegetation and soil are further promoted, thereby increasing SOC (Pang et al., 2020). Restoration measures such as grazing, reseeding, and fertilization affect soil microorganisms in the short term, which indirectly affects the decomposition of litter in soil and its corresponding nutrient contents (Bilotta et al., 2007; Drewry et al., 2008; Waring et al., 2022; Su et al., 2023). As a result, soil nutrients at the sites were higher under a relatively heavy grazing intensity. Furthermore, the “compensated growth” of grassland vegetation makes them absorb more nutrients from the soil under grazing conditions. Therefore, some of the soil nutrients are transferred to the ground for vegetation growth (Su et al., 2004; Peng et al., 2023). These results were also reflected in the study by Wang and De (2017), who found that reseeding restoration measures substantially increased the accumulation of TK, AP, and SOC in the soil in their study of the changes in soil nutrients in an alpine meadow under different supplementary sowing years. However, in the current study, the AN and AK contents at the reseeding and fertilizing sites were lower than those at the sites with different grazing intensities. Notably, this decrease might be caused by the increased interaction between the soil and vegetation due to reseeding and fertilization. Fertilization measures increase soil nutrients and promote vegetation growth. Simultaneously, reseeding greatly increased the aboveground vegetation, intensifying the soil nutrient consumption of plants. During the short repair years, the soil nutrients do not fully recover or reach a relatively stable state (Gao et al., 2019; Wang et al., 2023). The SOC content at the normal grazing sites was the lowest and increased to varying degrees at the other sites. This is probably because the decrease in grazing intensity followed by moderate grazing was conducive to soil nutrient accumulation and soil structure restoration in the grassland (Yang et al., 2018; Zhang et al., 2023). This concurs with the results obtained by Zhang et al. (2021) regarding the influences of grazing on soil properties in the eastern margin of the Qilian Mountains. Reseeding can affect the soil aggregate structure and enhance SOC sequestration (Liu et al., 2022). Fertilization can increase the SOC content by increasing the input of soil organic matter (Fang et al., 2012). Therefore, the SOC at the T4 restoration site was higher than that at the three different grazing and reseeding sites. The available nutrients at the T4 restoration site were higher than those at the T3 restoration site, which is in line with that of Hou and Guo (2021), who studied the dynamic response of plant nutrients to grazing intensity. The plant nutrient transfer rate differed under different grazing intensities, which resulted in an increase in available nutrients at the T4 restoration site (Hou and Guo, 2021).

### 4.2 Relationship between vegetation characteristics and soil factors

Different restoration measures have different effects on the soil properties and vegetation characteristics of grasslands (Keller et al., 2023). In grassland ecosystems, the physicochemical properties of soil are important factors that directly affect grassland vegetation. Therefore, understanding the correlation between soil factors and grassland vegetation characteristics provides a theoretical reference for the restoration of degraded grassland soils. Vegetation degradation is the primary cause of soil degradation and *vice versa* (Wu et al., 2020; Kooch et al., 2022).

Different disturbance conditions have different effects on the species structure, coverage, and biomass of grassland plant communities. Fertilization increases species diversity, enhances community competitiveness, promotes plant growth, and enhances soil nutrients (Zong et al., 2021). Our findings suggest that SOC and AK are two key factors affecting the vegetation characteristics of restored grasslands, and they have significant positive effects on grassland vegetation. SOC content is an important index for evaluating soil fertility. Reseeding measures can substantially promote SOC accumulation, and fertilization can also increase SOC content (Fang et al., 2012; Liu et al., 2022). In particular, the rate of organic carbon mineralization is relatively high within the first few years of fertilization (Lan et al., 2016). Under the comprehensive restoration measures at the T4 site, reseeding and fertilization increased the SOC content, promoted the absorption of nutrients by vegetation, and improved the grassland productivity (Dee et al., 2023). However, in short experimental years, the nitrogen and phosphorus contents in the soil were greatly affected by comprehensive factors, and the influences of fertilization on plant nutrients were greater than those on soil nutrients (Gao et al., 2019; Zong et al., 2021; Keller et al., 2023). At the T4 site, no Leguminosae, Cyperaceae, or inedible forage appeared, and the aboveground biomass of Gramineae, Forbs, edible herbage, and AGB was substantially higher than those under other treatments. An explanation for this result is the increased species richness and nutrient content of grasslands under relatively low grazing intensity, reseeding, and fertilization measures (Li et al., 2018), which promoted nutrient cycling between the vegetation and soil and asymmetric competition between vegetation and accelerated the growth of dominant species in grasslands (Donald, 1958; Hautier et al., 2015).

### 4.3 Challenges in grassland restoration

Our study investigated the short-term effects of different restoration measures combined with grazing intensity, reseeding, and fertilization on degraded grasslands. Most scholars have focused on the positive effects of artificial restorations (Feng et al., 2010; Wu et al., 2010; Zhang et al., 2020; Gu et al., 2022); however, there are also some negative effects. Improper timing and restoration techniques can also cause severe grassland degradation—for example, fertilization transforms underground plant competition into aboveground light competition (Donald, 1958; Niu et al., 2008), thereby reducing plant diversity (Harpole et al., 2016; Seabloom et al., 2020) and community stability (Song and Yu, 2015). The potential negative impact that fertilization could have on grasslands is dependent on the fertilizer application amount and fertilization time (Chen et al., 2013). Although no-till supplementary seeding causes less damage to grasslands than other supplementary seeding measures, it can be easily excluded by competition from native vegetation when the selection of supplementary pasture species is incorrect. This leads to a low species survival rate in no-till supplementary seeding, high improvement costs of supplementary seeding, poor stability of the grassland community, short service life of supplementary seeding grasslands, and other problems (Zhang et al., 2020). The combination of fertilization and reseeding can enhance grassland productivity and improve grassland conditions in the short term. However, the restoration succession of degraded grasslands is a very long process (Shang et al., 2017). Short-term studies can address productivity and livelihood issues but cannot explain the impact of restoration measures on vegetation diversity. Only long-term research can accurately evaluate the restoration of grasslands, and the corresponding restoration measures and models can be more reliable and convincing.

## 5 Conclusions

Based on the short-term restoration of degraded grasslands in an alpine meadow, reseeding and a combination of reseeding and fertilization effectively increased the SOC content but caused a decrease in available nutrients. Transferring livestock out of the pasture in advance, reseeding, and fertilization improved the height and coverage of grassland vegetation and markedly increased the grassland productivity. SOC was the main factor positively affecting the growth of grassland vegetation. Further restoration and management of degraded grasslands are needed to consider the sustainable development of grassland ecosystems. A long-term study should be conducted to select appropriate combinations of measures according to the local conditions. This should then be combined with a reasonable grassland management and utilization system to achieve the best restoration effects. The findings of this study may offer valuable theoretical insights for the ecological restoration of degraded grasslands and local development in alpine regions.

## Data availability statement

The raw data supporting the conclusions of this article will be made available by the authors, without undue reservation.

## Author contributions

YW: Data curation, Formal Analysis, Investigation, Methodology, Software, Visualization, Writing – original draft, Writing – review & editing. ZW: Data curation, Investigation, Methodology, Software, Visualization, Writing – review & editing. YK: Resources, Software, Visualization, Writing – review & editing. ZZ: Investigation, Resources, Writing – review & editing. DB: Investigation, Resources, Writing – review & editing. XS: Investigation, Resources, Writing – review & editing. JS: Conceptualization, Funding acquisition, Investigation, Project administration, Supervision, Validation, Writing – review & editing.
